# Physiological differences between a noncontinuous and a continuous endurance training protocol in recreational runners and metabolic demand prediction

**DOI:** 10.14814/phy2.13546

**Published:** 2017-12-15

**Authors:** Muhammad J. Ali, Balasekaran Govindasamay, Hoon Kay Hiang, Gerald Seet Gim Lee

**Affiliations:** ^1^ School of Mechanical & Aerospace Engineering Nanyang Technological University Singapore Singapore; ^2^ Physical Education and Sports Science, Human Bioenergetics Laboratory National Institute of Education Singapore Singapore; ^3^ Institute for Sports Research Nanyang Technological University Singapore Singapore

**Keywords:** Critical speed, endurance performance, lactate prediction, physiological differences, recreational runners

## Abstract

This study investigated the physiological difference in recreational runners between a noncontinuous and a continuous endurance training protocol. It also aimed to determine physiological surrogate that could monitor metabolic demand of prolonged running in real‐time. For data collection, a total of 18 active male recreational runners were recruited. Physiological (HR, RR, RER,* Ṽ*O_2_, BLa), and overall perceptual (RPE_O_) responses were recorded against three designed test sessions. Session 1 included *Ṽ*O_2submax_ test to determine critical speed (CS) at anaerobic threshold (AT). Session 2 was the noncontinuous CS test until exhaustion, having 4:1 min work‐to‐rest ratio at CS, whereas session 3 was the continuous CS test till exhaustion. As 1‐min recovery during session 2 may change fatigue behavior, it was hypothesized that it will significantly change the physiological stress and hence endurance outcomes. Results reported average time to exhaustion (TTE) was 37.33(9.8) mins for session 2 and 23.28(9.87) mins for session 3. Participants experienced relatively higher metabolic demand (BLa) 6.78(1.43) mmol.l^−1^ in session 3 as compared to session 2 (5.52(0.93) mmol.l^−1^). RER was observed to increase in session 3 and decrease in session 2. Student's paired *t*‐test only reported a significant difference in TTE, ṼO_2_, RER, RPE_O_, and BLa at “End” between session 2 and 3. Reported difference in RPE_O_ and %HR_max_ at “AT” were 5 (2.2) and 89.8 (2.60)% during session 2 and 6 (2.5) and 89.8 (2.59)% during session 3, respectively. Regression analysis reported strong correlation of %HR_max_ (adj. R‐square = 0.588) with BLa than RPE_O_ (adj. R‐square = 0.541). The summary of findings suggests that decreasing RER increased TTE and reduced BLa toward “End” during session 2 which might have helped to have better endurance. The %HR_max_ was identified to be used as a better noninvasive surrogate of endurance intensity estimator.

## Introduction

During exercise, the cardiorespiratory system is subject to supply a continuous flow of oxygen and nutrients to the skeletal muscles and to remove metabolic products for cellular respiration (Myers 2001; Crisp et al. 2013). During endurance exercise, the physiological response is dependent on the running speed. Literature has reported a strong correlation (0.68–0.98) of endurance pace with critical speed (CS) at anaerobic threshold (AT = blood lactate of 4.0 mmol.L^−1^) over the long‐distance competitions (Faude et al. 2009). Sports scientists have proposed various methods to determine CS that could best represent the optimal endurance speed. Some reported methods include linear regression of distance versus time, the speed at anaerobic threshold and associated maximum lactate steady state (MLSS) speed (Kranenburg and Smith 1996; Smith and Jones 2001; Bull et al. 2008). To evaluate physiological response, the critical speed (CS) was selected in this research, considering the fact that treadmill CS may overestimate the endurance performance (Kranenburg and Smith 1996).

Majority of the endurance studies in the literature are focused on highly trained distance runners or elite runners (Saunders et al. 2004; Seiler and Sjursen 2004; Joyner and Coyle 2008; de Lucas et al. 2012; Penteado et al. 2014). However, this study focused to evaluate the physiological response in recreational runner during a noncontinuous and continuous endurance protocol. Such investigation will help to examine whether existing findings on endurance performance in the trained runner (Penteado et al. 2014) are directly productive to active recreational runners or not. Furthermore, the noncontinuous running protocols (intermittent stage protocol) are quite popular in term of their cardiovascular benefits, aerobic fitness, metabolic, and skeletal muscle adaptations in comparison with the continuous run (Helgerud et al. 2007; Perry et al. 2008; Hottenrott et al. 2012). It is important to observe their physiological response, TTE, BLa, and RPE_O_ response with endurance fatigue development.

To achieve the optimal endurance response for the physiological system (Seiler and Sjursen 2004; Seiler and Hetlelid 2005) and valid perceptual response while running at the known CS of LT4 (Santos‐Concejero et al. 2013), the noncontinuous test had a magnitude of 4:1 min (work: rest) ratio. The 4‐min running stage was considered as an optimal duration to elicit high physiological load in relation to endurance pace (Seiler and Sjursen 2004). Whereas, 1‐min rest was selected due to its limited impact on recovery from the physiological stress (Seiler and Hetlelid 2005). The continuous test was believed to optimally stress the physiological system and hypothesized that it may undermine the maximum lactate steady state (MLSS) response, as observed by Penteado et al. (2014) for trained endurance runners. It was believed that comparative analysis between both tests will help to understand the physiological difference. The physiological analysis may also provide a noninvasive means of estimating metabolic demand, either through a valid perceptual variable (RPE_O_) or any other noninvasive measurable physiological variable.

To the best of our knowledge, no study has investigated the acute physiological response of recreation runners during a noncontinuous and continuous test in a comparative fashion. Based on the evidence from the tested studies for trained distance runners (de Lucas et al. 2012; Penteado et al. 2014), this study hypothesized that 1‐min recovery will lead to MLSS response during the noncontinuous test for recreational runners. The second hypothesis was that physiological stress development due to endurance fatigue will be different between the noncontinuous and continuous test and will result in a difference in endurance performance (TTE, BLa at “End”). Furthermore, as RPE_O_ was found to be changing without any change in lactate level in highly trained runners at intensities related to MLSS (Seiler and Sjursen 2004), one of the objectives was to determine the noninvasive physiological surrogate of metabolic demand in recreational runners.

## Method

### Subjects

The 18‐healthy active male recreational runners (age 23.05 (1.51) years, BMI 22 (1.98), *Ṽ*O_2max_ 57.45 (5.35) mL·kg^−1^·min^−1^, HR_max_ 190.66 (7.70) beats·min^−1^, % body fat 12.87 (3.26)) gave written informed consent to participate in this study. The study got approval from ethical committee “Institutional Review Board, Nanyang Technological University”, Singapore. The exclusion criteria of the study include *Ṽ*O_2max_ < 50 mL·kg^−1^.min^−1^ and >65 mL·kg^−1^·min^−1^, running duration <30 min, running frequency <2 times a week, BMI > 25 or any lower extremity muscle or bone injury. Participants were instructed to restrain from any hard exercises, gym or running training habits during the last 36 h before the test session. Participants were also instructed to remain hydrated and refrain from any intake of meal, caffeine or energy drink on the test day, before the start of the test.

### Experimental design

Tests were conducted under laboratory‐controlled conditions at the same hours of the day to avoid any circadian variation on oxygen kinetics during running (Carter et al. 2002). Participants were instructed to wear the same running shoes and clothes to avoid external factors affecting running economy (Fuller et al. 2015). Resting BLa level (<1.5 mmol·L^−1^) (Goodwin et al. 2007; van Hall 2010) was tested to ensure that BLa levels were at the baseline before the start of each test. Anthropometric measurements (height, body mass, and percentage body fat, etc.) were taken using “InBody 720 body composition analyzer.” RPE‐OMNI scale (Irving et al. 2006) was introduced to the participants to monitor overall perceived exertion (RPE_O_). For cardio‐respiratory data collection, “Parvo Medics True One 2400” metabolic analyzer was calibrated using standard gas calibration method (with ±1% error tolerance) and flow meter calibration methods (with ±1% error tolerance). Gas calibration was performed by releasing the E‐cylinder gas (mixture contained 16% O_2_ and 4.05% CO_2_) in the 4‐L gas mixing chamber to use as a comparative standard, which was based on the temperature (22°C), humidity level (72%), and barometric pressure (752 mmHg) of the room. The flow calibration used a 3‐L syringe to simulate a breathing flow rate as a comparative standard to the participants’ breathing. A total of four flushes and five strokes were recorded by the system and system calibration was accepted only when the calibrated error was less than 1%. Following system calibration, Polar chest strap sensor was integrated to record heart beats in real‐time. For BLa concentration, a sample size of 50 *μ*L was collected in a 100 *μ*L tube using the finger pricking device. Sample aspiration of only 25 *μ*L was used by the YSI 2900 analyzer to measure BLa in the body in relation to running intensity and fatigue.

After all initial preparations, participants were asked to do warm‐up for 5–10 min at 8–8.5 km·h^‐1^ on the WOODWAY treadmill, followed by quick leg stretching exercise to get ready for the final testing phase during that session.

### Experimental protocol

A total of three testing sessions: (Session 1) progressive submaximal treadmill run test, (Session 2) noncontinuous CS run to volitional exhaustion, (Session 3) continuous CS run to volitional exhaustion, were conducted. Session 2 and 3 were conducted in listed order and recovery period between each test was 5–7 days.

#### Session 1

A progressive submaximal treadmill run (*Ṽ*O_2submax_) test (starting from 10 km·h^−1^ and 1% gradient with an increment of 1 km·h^−1^ till anaerobic threshold, having 4:1 min work to rest ratio) was conducted on “WOODWAY” motorized treadmill. A gradient of 1% was used to equate the energy cost of outdoor running on the treadmill (Jones and Doust 1996). Blood samples were collected and analyzed to determine CS (Sjödin and Jacobs 1981; Santos‐Concejero et al. 2013), using the spline curve estimation method between the treadmill speed and BLa response. Following 30‐min rest after completion of *Ṽ*O_2submax_ test, Astrand‐modified running maximal oxygen consumption test (starting speed = 10 km·h^‐1^, gradient = 1%, speed increment = 1 km·h^‐1^ after every 1‐min till treadmill speed of 15 km·h^‐1^, 2% gradient was increased after every minute once treadmil speed reached 15 KPH) (Ali et al. 2014) was conducted to determine the *Ṽ*O_2max_ and HR_max_. The maximal effort was verified by a plateau in *Ṽ*O_2_, respiratory exchange ratio (RER) >1.1, heart rate in the range of 95% of maximal heart rate (220‐age) (Howley et al. 1995).

#### Session 2

A noncontinuous CS run to volitional exhaustion protocol (work: rest = 4:1 min, constant CS of each participant and gradient = 1%) was administered. Cardio‐respiratory data were collected throughout the test. RPE_O_ data were recorded during the last 1‐min of each active stage. Blood samples were collected during the resting intervals. The test was terminated when individual's RPE_O_ reached 10.

#### Session 3

A continuous CS run to volitional exhaustion protocol (constant CS till volitional exhaustion, gradient = 1%) was administered. Cardio‐respiratory data were monitored throughout the test. RPE_O_ data were recorded after every 2‐min until RPE_O_ reached 8, followed by every 1‐min till RPE_O_ = 10. Only two blood samples were collected from finger, one at the start and one at the termination of the test to determine the maximum BLa.

### Data processing and statistical analysis

During session 1, CS was determined using spline curve estimation function between treadmill speed and BLa response, followed by *Ṽ*O_2max_ test to determine *Ṽ*O_2max_ and HR_max_. For physiological measurements during session 2 and 3, “5 breath averaging window” respiratory data were averaged using the 1‐min averaging window. Physiological data during session 2 were extracted during the last minute of each 4‐min active stage. During session 3, physiological data were monitored throughout the test. Furthermore, data for all the physiological variables, RPE_O_, and BLa were plotted against percentage completion time (% completion time) to determine the underlying function (using power estimation function) to represent the variable adjustment along the course of fatigue run to volitional exhaustion.

For identifying most significant physiological variables, Kendal tau_b correlation with a significance level of 95% was used. Paired sample *t*‐test with a significance level of 95% was used to determine the difference in physiological, perceptual, and endurance variables between noncontinuous and continuous test. Furthermore, regression analysis was performed to determine a most responsive parameter that could predict BLa concentration. Statistical analysis was carried in SPSS 20 and OriginPro 9.0.

## Results

The maximum aerobic capacity (*Ṽ*O_2max_) and maximum heart rate (HR_max_), obtained from session 1, were 57.45 (5.35) mL·kg^−1^·min^−1^ and 191 (8.0) beats·min^−1^, respectively. CS for the selected group of participants was 13.14 (1.47) km ·L^−1^, and %HR_max_ was 89.81 (2.6). Average TTE was 37.33(9.80) mins for noncontinuous and 23.28 (9.87) mins for the continuous test. Reported TTE at CS in this study was almost similar to Penteado's elite group of runner (Penteado et al. 2014). Statistics (mean (SD)) for TTE and physiological variables (*Ṽ*O_2,_ HR, %HR_max_, RR, RER, RPE_O_, and BLa) at “AT,” and “End” stages of the noncontinuous and continuous test are summarized in Table 1.

**Table 1:** Individual's response data include TTE, HR, RR, RER, and RPE_O_ at “AT” and “End” stage during the noncontinuous CS and continuous CS test

Endurance variables	Stages	Noncontinuous test	continuous test	Paired sample *T*‐test	
TTE (min)		37.33 ± 9.8	23.28 ± 9.87	a	*t* = 7.179, *P* = 0.0001
ṼO_2_ (mL·kg^−1^·min^−1^)	AT	46.94 ± 4.9	47.58 ± 5.57		*t* = −1.830, *P* = 0.085
	END	48.12 ± 4.88	49.73 ± 5.57	a	*t* = −3.063, *P* = 0.007
HR (beats·min^−1^)	AT	171 ± 7.0	171 ± 7.0		*t* = 0.669, *P* = 0.513
	END	179 ± 8.0	181 ± 8.0		*t* = −1.638, *P* = 0.120
%HR_max_	AT	89.81 ± 2.60	89.81 ± 2.60		*t* = 0.740, *P* = 0.469
	END	94.06 ± 1.72	94.88 ± 2.19		*t* = −1.605, *P* = 0.127
RR (min^−1^)	AT	48.0 ± 8.0	48.0 ± 8.0		*t* = 0.225, *P* = 0.825
	END	58.0 ± 8.0	58.0 ± 8.0		*t* = −0.550, *P* = 0.589
RER	AT	0.98 ± 0.04	0.99 ± 0.04		*t* = −1.463, *P* = 0.162
	END	0.97 ± 0.04	1.0 ± 0.05	a	*t* = −4.024, *P* = 0.001
RPE_O_	AT	5.0 ± 2.20	6.0 ± 2.5		*t* = −1.681, *P* = 0.111
	END	9.4 ± 0.85	9.90 ± 0.24	a	*t* = −3.007, *P* = 0.008
Bla (mmol·L^−1^)	END	5.52 ± 0.93	6.78 ± 1.43	a	*t* = −4.311, *P* = 0.0001

Paired *t*‐test with significance level of 95% showed the difference in endurance variables between both tests. TTE, time to exhaustion; *Ṽ*O_2_, oxygen cost of running; HR, Heart Rate; %HR_max_, heart rate intensity; AT, anaerobic threshold; End, termination; RR, respiratory rate; RER, respiratory exchange ratio; RPE_O_, overall rated perceived exertion; BLa, blood lactate.

a 95% confidence interval significance (two‐tailed).

Participants experienced relatively higher BLa (6.78(1.43) mmol·L^−1^) in continuous test than in noncontinuous test (5.52 (0.93) mmol·L^−1^). RER was found to be significantly increasing during the continuous test in comparison with the noncontinuous test. Participants felt more exhausted at “End” stage in the continuous test in comparison to the noncontinuous test for the overall body feel. Summary of the identified significant variables is reported in Table 1.

**Figure 1:**
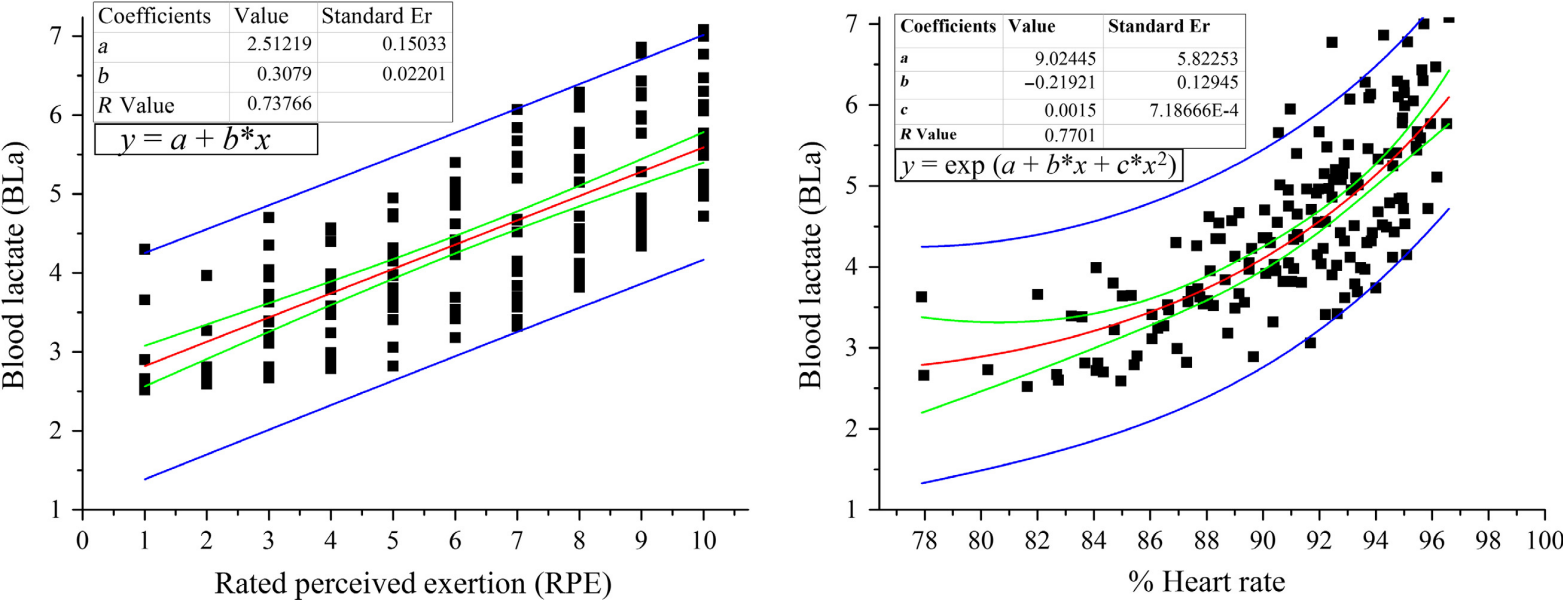
Linear (on left) and exponential (on right) regression analysis for predicting BLa concentration based on RPE_O_ and %HR_max_, respectively. The red line represents the fitted curve, whereas two green lines show 95% confidence range and blues lines represent 95% prediction range.

Kendal tau_b reported a significant correlation of RPE_O_ with *Ṽ*O_2_, HR, RR, and BLa, whereas %HR_max_ had a stronger correlation with HR, RPE_O_, and BLa in both tests, as indicated in Table 2. To determine noninvasive surrogate of metabolic demand, regression analysis of a noncontinuous test (shown in Fig. 1) reported that BLa concentration was found to be more responsive to the change in %HR_max_ (adj. *R* ^2^ = 0.588) than RPE_O_ (adj. *R* ^2 ^= 0.541), and residual RMS error was also less (0.677, 0.714, respectively). Using these model, when BLa was predicted in session 3, mean (SD) error at “End” was also found to be higher for RPE_O_ (1.19 (1.43)) than %HR_max_ (1.26 (0.96)).

**Table 2:** Correlation of physiological variables with RPE_O_ and %HR_max_ using Kendall's tau_b statistical significance

Kendall's tau_b correlation			ṼO_2_	HR	RR	RER	RPE_O_	BLa
Noncontinuous test	RPE_O_	Correlation coefficient	0.246^a^	0.435^a^	0.368^a^	−0.063	1.000	0.573^a^
		Sig. (two‐tailed)	0.000	0.000	0.000	0.251		0.000
	%HR_max_	Correlation coefficient	0.110^b^	0.482^a^	0.234^a^	−0.025	0.601^a^	0.580^a^
		Sig. (two‐tailed)	0.035	0.000	0.000	0.627	0.000	0.000
Continuous test	RPE_O_	Correlation coefficient	0.251^a^	0.475^a^	0.393^a^	0.041	1.000	–
		Sig. (two‐tailed)	0.000	0.000	0.000	0.459		–
	%HR_max_	Correlation coefficient	0.155^a^	0.529^a^	0.345^a^	0.200^a^	0.666^a^	–
		Sig. (two‐tailed)	0.003	0.000	0.000	0.000	0.000	–

The “Kendall's tau_b” correlation significance of several physiological variables with RPE_O_ and %HR_max_. The shaded area shows the significant association of %HR_max_ with RPE_O_ and BLa.

a Correlation is significant at the 0.01 level (two‐tailed).

b Correlation is significant at the 0.05 level (two‐tailed).

## Discussion

Critical speed (CS) has been investigated, in literature, to show the similarity with the maximal lactate steady state (MLSS) intensity during cycling (Oyono‐Enguelle et al. 1990) and running (Föhrenbach et al. 1987; Smith and Jones 2001). The pioneering study (Moritani et al. 1981) had suggested that the CS is an intensity which could be maintained for a very long time. However, subsequent studies on continuous exercise at this intensity, have shown a systematic increase in BLa concentration and times to exhaustion range between 20 and 40 min (Brickley et al. 2002; Bull et al. 2008). From this point of view, CS can be characterized as the boundary between steady state (below) and nonsteady state (above) intensity (Vanhatalo et al. 2011; De Lucas et al. 2013). Because of its practicality, the critical speed test has become widely acceptable as an index of endurance‐specific performance for trained endurance runners (Florence and Weir 1997). However, for recreational runners in this study, CS was hypothesized to achieve same metabolic relation (MLSS) with endurance performance as of endurance runners during the noncontinuous and continuous test.

This study also investigated the physiological difference in endurance running between a noncontinuous and a continuous test for recreational runners. CS represented the endurance pace during both tests. The selected 4‐min active stage duration during the noncontinuous test resulted in optimal cardiorespiratory response (Seiler and Sjursen 2004; Penteado et al. 2014). A 1‐min rest was subject to provide an imbalance in intracellular restitution and maintenance of O_2_ kinetics and to replicate lactate steady state conditions (Seiler and Hetlelid 2005; Penteado et al. 2014).

Participants in this research experienced less metabolic demand during the noncontinuous test as compared to continuous test, probably due to better decreasing RER trend observed in this study. BLa concentration at 12th minute (3.99 (0.64) mmol·L^−1^) and at the “End” stage of the test (5.52 (0.93) mmol·L^−1^) were reported to be in the range of 0.5–2 mmol·L^−1^. Such variation in BLa concentration met the criterion of 1 mmol·L^−1^ (Beneke 2003) to determine MLSS response. Only five participants among 18 runners showed BLa increase higher than 2.0 mmol·L^−1^ and can be considered that these participants were running at a very close pace to MLSS (de Lucas et al. 2012). Such finding suggests that selected CS resulted in MLSS response, as of endurance pace. Whereas, continuous CS running overestimated the MLSS, similarly reported by Smith and Jones (2001), Penteado et al. (2014) and Vanhatalo et al. (2011). Only four participants meet the criterion of 1 mmol·L^−1^ to determine maximal lactate steady state response (Beneke 2003), whereas the majority of the participants were found to increase the lactate concentration higher than 2 mmol·L^−1^ from the MLSS. Therefore, it was considered that continuous test resulted in higher BLa in recreational runners during fatigue progression and may undermine their MLSS response at CS.

The observed increase in TTE in recreational runners during noncontinuous running (~1.6 times as compared to continuous TTE) suggests that noncontinuous protocol is more productive to expose working muscles for a long duration under the same cardio‐respiratory stress levels at CS. The observed difference in TTE in recreational runners was also found to be in close proximity with distance trained runners (~2 times of continuous TTE), studied by Penteado et al. (2014). Furthermore studies are needed to understand what causes the change in TTE under such testing conditions while considering the responses of individual training speed, personalized energy contribution models, training history, muscle mass composition, aerobic fitness, and recovery dynamics during endurance speed run (Ramos‐Jiménez et al. 2008; Edgett et al. 2013). Considering the physiological over studied variables between session 2 and 3, this study suggests that TTE may subject to opposing trend in RER, reduced running economy, increased RPE_O_ and BLa. RER was subject to increase in continuous test, whereas opposing trend was observed during the noncontinuous test. Participants were also more physiologically stressed as consumption of *Ṽ*O_2_ was higher in continuous test in comparison with the noncontinuous test. Participant also experienced relatively higher BLa and RPE_O_ toward “End” stage during the continuous test.

**Figure 2:**
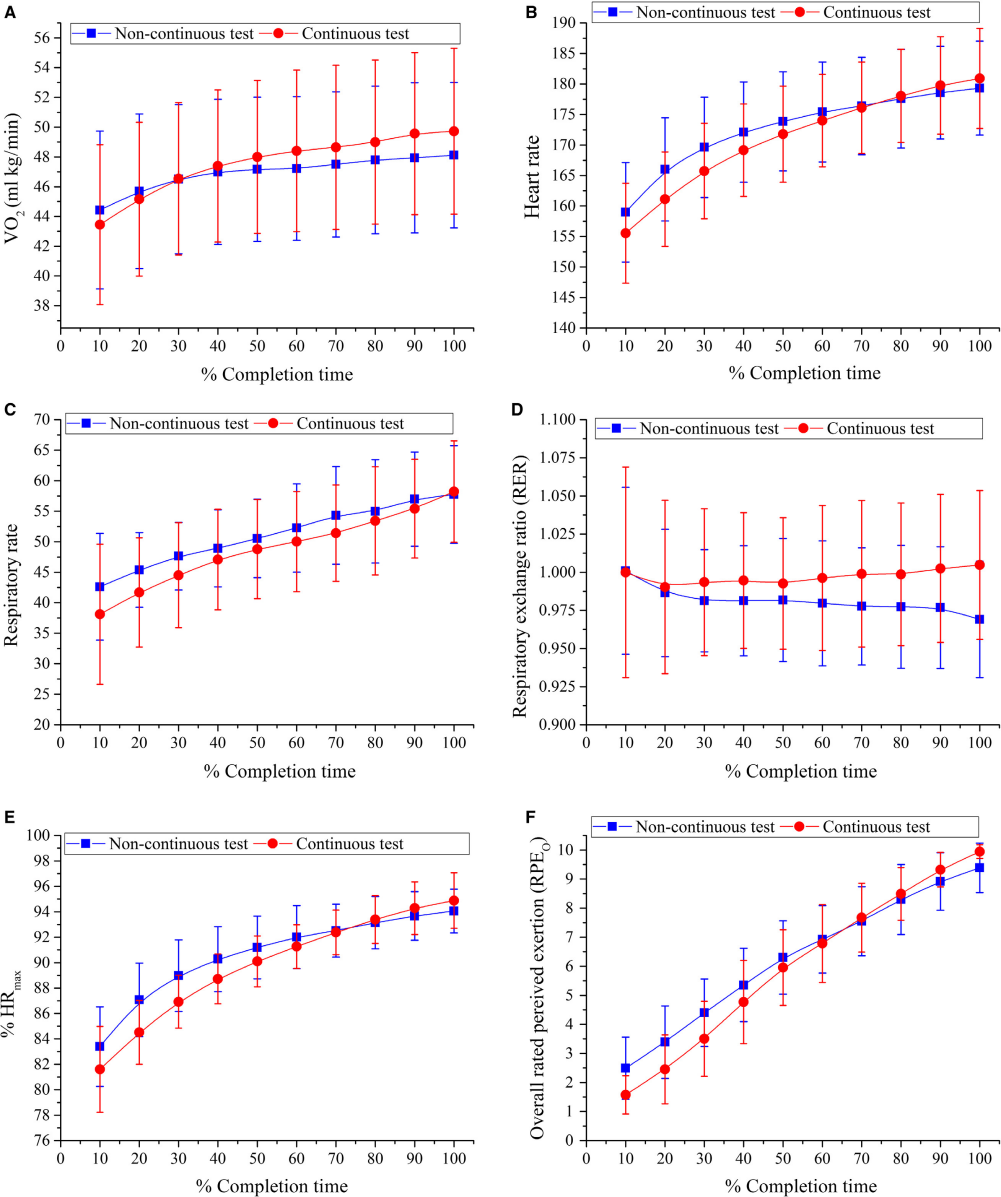
Physiological response (ṼO_2_ (A), HR (B), RR (C), RER (D), %HR_max_ (E)) and RPE_O_ (F) against % completion time (%TTE) during the noncontinuous and continuous test. All values are reported as mean ± SD (s) at every 10% of TTE.

The trend (mean (SD)) for the physiological and perceptual variables (*Ṽ*O_2_, HR, RR, RER, %HR_max,_ and RPE_O_) during a noncontinuous and a continuous test are shown in Figure 2. Selected CS reported sufficient cardiorespiratory stress on the physiological system as no difference was found in HR, RR and %HR_max_ at End stage between the noncontinuous and continuous CS test. A significant difference was found in exercise tolerance (TTE), *Ṽ*O_2,_ RER, RPE_O_, and BLa between both selected protocols.

**Figure 3:**
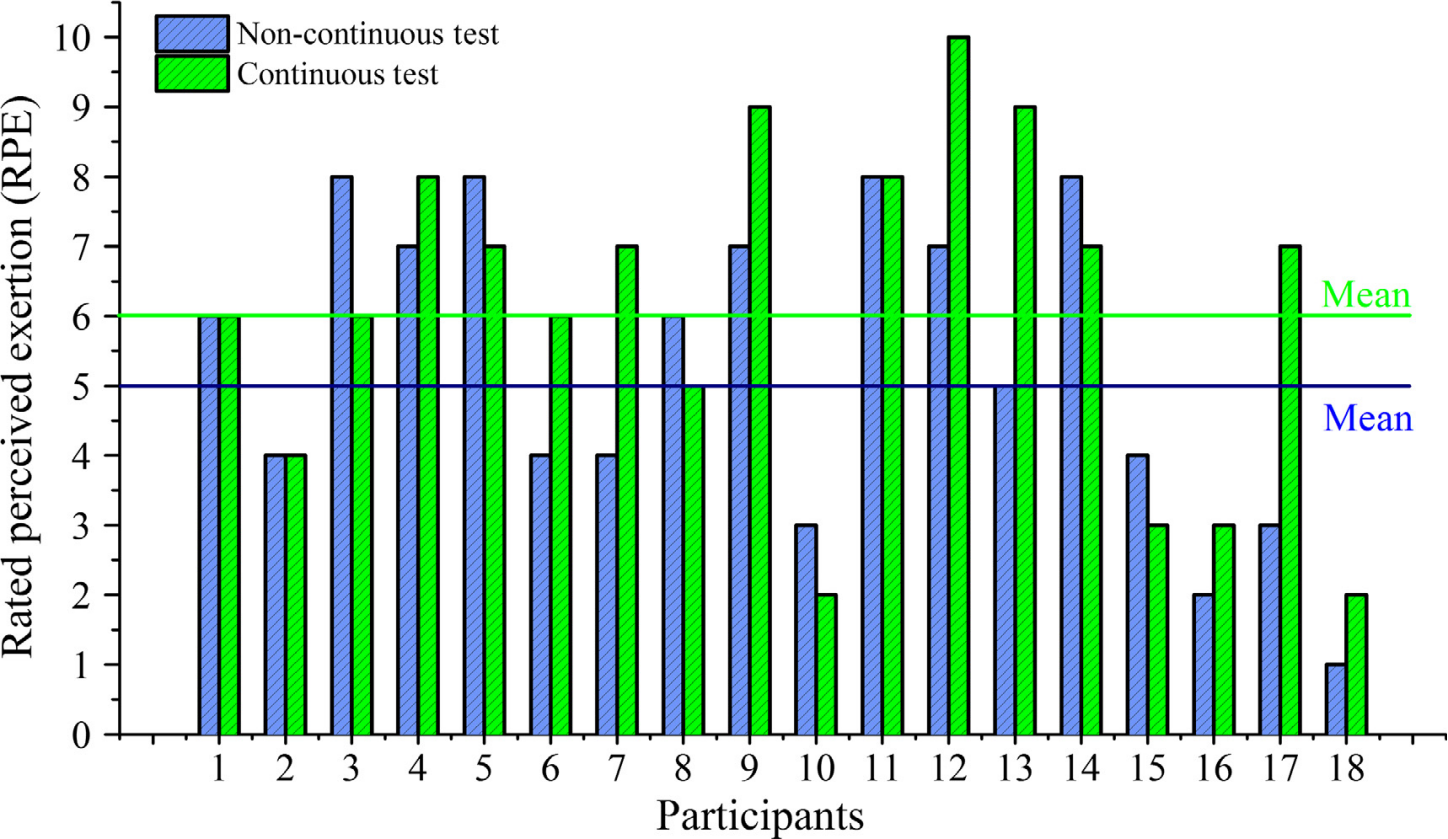
Rated perceived exertion at anaerobic threshold (AT) for the noncontinuous and continuous test.

Individuals’ perceived exertion (RPE_O_) in relation to the sustained endurance time showed that individuals reached “AT” at RPE_O_ of 5 (2.2) (40 (23.8) % of TTE) during the noncontinuous test and of 6 (2.5) (52.7 (21.35) % of TTE) during the continuous test. The difference in reported RPE_O_ among individuals was subject to BLa rise. Individuals, whose BLa rose faster towards AT in response to sustained time at CS, reported RPE_O_ less than 5 during the noncontinuous test and 6 during the continuous test. Similarly, for those individuals whose BLa response delayed toward AT against sustained time at CS, their RPE_O_ was higher than the mean value during both tests. The observed response kinetics between BLa and RPE_O_ may give rise to the tendency of under or overestimating the associated metabolic demand with RPE_O_ 5 during the noncontinuous test and RPE_O_ 6 during the continuous test. The difference in reported RPE_O_ at AT for both tests are shown in Figure 3. As different evidence do exist regarding the relation between RPE_O_ and lactate, summarized by Mihevic (1980), the validity of using RPE_O_ as a remote parameter to determine AT related intensity is a serious concern. To better predict BLa during constant CS protocol, %HR_max_ is proposed in this research. Furthermore, the correlation between %HR_max_, RPE_O_, and BLa are shown in Table 2. Regression analysis has proposed an exponential best‐fit model, shown in Figure 1, to predict BLa concentration for %HR_max_ in the range of 78–100%.

To our knowledge, a study by Penteado et al. (2014) has partially explained similar research questions on endurance trained runners. Penteado's study was conducted outside laboratory without physiological data evidence. Whereas, this research study includes active recreational runners and has explained the research questions with physiological data evidence. This research study also has limitations. Limitations include subject motivation, diet, individual's sports training history and fitness level, laboratory conditions, treadmill surface and running shoes, the inter‐individual difference in energy kinetics, muscle mass composition, and many more.

## Conclusion

The results of present study establish that critical speed (CS) was associated with MLSS during the noncontinuous test (probably because of one‐minute recovery) whereas continuous test overestimated the MLSS. Endurance time during the noncontinuous test was almost 1.6 times higher than the continuous test, due to 1‐min rest and decreasing trend in RER. No difference was found in cardiorespiratory stress at the time of exhaustion, whereas individuals’ metabolic demand was higher during the continuous test. The RPE_O_ was found to be changing linearly and could be used to monitor sustained time BLa, whereas %HR_max_ was found to show stronger correlation and less prediction error than RPE_O_. The findings suggest that %HR_max_ may be a better parameter to be used to determine BLa in future. This knowledge is helpful for the coaches and recreational runners to help them plan their endurance training sessions and monitor their metabolic demand against endurance stress noninvasively through %HR_max_.

## Conflict of Interest

Authors have no conflict of interest.
