# Physiological differences between a noncontinuous and a continuous endurance training protocol in recreational runners and metabolic demand prediction

**DOI:** 10.14814/phy2.13607

**Published:** 2018-02-07

Muhammad J. Ali, Govindasamy Balasekaran, Hoon Kay Hiang, Gerald Seet Gim Lee

*Physiol Rep, 5 (24), 2017, e13546,* https://doi.org/10.14814/phy2.13546

In Muhammad et al. (2017), the second author's name was misspelled as “Balasekaran Govindasamay” and should instead be “Govindasamy Balasekaran”.

The authors apologise for the error.
